# Empowering the nursing workforce for cyber-resilience: Strategic preparedness and care management during hospital cyberattacks

**DOI:** 10.1371/journal.pdig.0001291

**Published:** 2026-03-27

**Authors:** Amir Mohammad Dorosti, Amin Soheili, Hamed Gholizad Gougjehyaran, Mir Amirhossein Seyednazari

**Affiliations:** 1 Student Research Committee, Khoy University of Medical Sciences, Khoy, Iran; 2 Department of Nursing, Khoy University of Medical Sciences, Khoy, Iran; 3 Student Research Committee, Tabriz University of Medical Sciences, Tabriz, Iran; University of Wurzburg: Julius-Maximilians-Universitat Wurzburg, GERMANY

## Introduction

Healthcare facilities are now ideal targets for cyberattackers due to their growing reliance on electronic systems in the digital age [1]. These attacks can have disastrous effects on the health system, interfere with patient care, and jeopardize patient safety [2].

Any malevolent attempt to compromise a medical facility’s information technology (IT) systems is referred to as a cyberattack in the field of medicine. These assaults can range from social engineering and phishing (suspicious emails used to get login credentials) to destructive ransomware attacks that encrypt vital data. Clinical professionals, especially nurses, are the major stakeholders in ensuring patient safety when these technologies malfunction, even while IT security teams oversee the digital infrastructure. Nurses frequently lack the formal training necessary to switch from digital to manual care practices during such operational problems, despite their position as the “human firewall.”

Medical facilities have increasingly become the target of ransomware attacks and data breaches. Significant events, such as the WannaCry assault, demonstrate the extensive operational ramifications [1]. Ransomware can cause treatment delays and patient suffering by blocking access to imaging systems, medical equipment, and Electronic Medical Records (EMRs) [3]. This necessitates long recuperation programs and a return to analog processes.

### Perspective approach and scope

We used phrases such as “Nursing,” “Cybersecurity,” “Hospital Cyberattacks,” and “Patient Safety” in a focused literature search across PubMed, CINAHL, and Google Scholar to develop this viewpoint. Recent (2019–2025) peer-reviewed publications were given priority, including technical reports on significant breaches like WannaCry, simulation-based research on threat detection, and qualitative studies on nurse experiences. Our practical recommendations are based on this synthesis of multi-level evidence.

## The preparedness gap: Awareness versus action

As the front line of healthcare, nurses play a critical role in responding to cyber disasters. Research, however, shows a significant disconnect between practice and awareness. Although most nurses are aware of cyber threats, they often lack the confidence and practical skills to handle such situations [4].

Forty percent of the 20 ICU nurses in one simulated research were unable to recognize the hack at all [5]. This indicates a serious threat-detection error. Nurses’ self-confidence in identifying and reporting cyber events was poor, despite their awareness scores being satisfactory, according to a different multi-center study. This disconnection between theoretical understanding and practical skills makes it impossible to take quick, efficient preventative measures [6].

## Real-world impact on patient care

The dire repercussions are demonstrated by actual occurrences. Treatments were delayed, and access to information systems was cut off as a result of the WannaCry attack on the UK’s NHS [7].

A cyberattack’s effects go much beyond making EMRs unavailable. It essentially transforms a single-institution incident into a regional catastrophe that requires care diversion to nearby hospitals by paralyzing Pharmacy Information Systems (PIS), causing medication administration errors; disrupting Laboratory Information Systems (LIS) and Radiology (PACS), delaying vital diagnostics; and stopping scheduling and billing systems.

According to qualitative research, frequent interruptions impede diagnostic services and put frontline employees under a great deal of operational and psychological strain [8]. The need for multi-layered communication technologies and strong downtime preparations is highlighted by the breakdown of EMR and WLAN-based communication paths during attacks [9].

## Deficiencies in training and knowledge

The lack of focused, hands-on instruction in nursing education is regularly demonstrated by analyses [10]. Research shows that nurses have poor cyber-risk awareness. Curricula need to be upgraded to incorporate practical skills such as secure operations, threat assessment, and disruption response because current training is inadequate [11].

Implementing secure behaviors is significantly hampered by human factors, such as employee burnout and infrequent training [12]. To support best practices, straightforward tools such as the “LOCK” (Log-off, Observe, Check, Keep) checklist have been suggested [13].

## Impact on patient safety and care delivery

Patient safety is directly threatened by digital interruptions, which result in recorded treatment delays and restricted access to test data. Additionally, the psychological strain on employees is enormous. According to qualitative research, “cyber shock” among employees might worsen burnout and affect clinical judgment. Critical decision-making and team communication may be hampered by this stress [2,11,12].

## Actionable recommendations

To improve nurses’ cyber-resilience, specific, implementable improvements are required. We recommend the following priorities:

1. **Execute Multi-Vector Simulations:** Hospitals should often exercise phishing and social engineering simulations in addition to system outages. Through these activities, nurses may practice the immediate reporting chain and identify misleading communications, which are the most prevalent way that ransomware enters a system [9].
2. **Institutionalize ‘Cyber Hygiene’ as a Core Competency:** Hospital orientations and nursing curriculum need to incorporate practical skills in addition to theory. Strong password management (never sharing passwords, following scheduled password changes, and implementing multi-factor authentication), device safety (forbidding the use of unknown USB drives on work computers), data protection (encrypting folders holding sensitive data), and stringent session security (turning off systems when not in use) are some examples of these particular competencies [10].
3. **Elevate Cyber Incidents to Hospital Emergency Codes:** Cyberattacks are more than just technical errors. In order to initiate an instant switch to manual treatment procedures, hospital administration needs to include cyber-response into the emergency management framework by creating a unique “Hospital Emergency Code” (such as Code Silver or Code Cyber) [14].
4. **Strengthen Communication Infrastructure:** Create offline, multi-layered communication solutions to keep teams coordinated if principal networks malfunction [15].
5. **Promote Interdisciplinary Collaboration:** To implement all-encompassing security methods, form collaborative teams of nurses, IT specialists, administrators, and cybersecurity specialists [3,12].
6. **Offer Staff Support:** To address staff fatigue and the psychological suffering (sometimes known as “cyber shock”) that follows cyber incidents, implement mental health support programs [5,6].

In conclusion, hospital security breaches are a complicated clinical emergency that requires more than just technological fixes. This viewpoint emphasizes that although nurses have a basic understanding, there is still a big gap in their actual readiness for managing care in the event of digital failures. Healthcare systems may guarantee patient safety in an increasingly fragile digital age by adopting a human-centric paradigm, which includes addressing cyber catastrophes as regional disasters and integrating cyber hygiene into fundamental nursing competencies.
